# Flame Retardant Polypropylene Composites with Low Densities

**DOI:** 10.3390/ma12010152

**Published:** 2019-01-05

**Authors:** Nerea Pérez, Xiao-Lin Qi, Shibin Nie, Pablo Acuña, Ming-Jun Chen, De-Yi Wang

**Affiliations:** 1 IMDEA Materials Institute, C/Eric Kandel, 2, 28906 Getafe, Madrid, Spain; nerea.perez@gmail.com (N.P.); xiaolin.qi@imdea.org (X.-L.Q.); nsb@mail.ustc.edu.cn (S.N.); pablo.acuna@imdea.org (P.A.); 2 School of Mining and Safety Engineering, Anhui University of Science and Technology, Huainan 233100, China; 3 School of Science, Xihua University, Chengdu 610039, China

**Keywords:** polypropylene, flame retardancy, zirconium phosphate, low-density

## Abstract

Polypropylene (PP) is currently widely used in areas requiring lightweight materials because of its low density. Due to the intrinsic flammability, the application of PP is restricted in many conditions. Aluminum trihydroxide (ATH) is reported as a practical flame retardant for PP, but the addition of ATH often diminishes the lightweight advantage of PP. Therefore, in this work, glass bubbles (GB) and octacedylamine-modified zirconium phosphate (mZrP) are introduced into the PP/ATH composite in order to lower the material density and simultaneously maintain/enhance the flame retardancy. A series of PP composites have been prepared to explore the formulation which can endow the composite with balanced flame retardancy, good mechanical properties, and low density. The morphology, thermal stability, flame retardancy, and mechanical properties of the composites were characterized. The results indicated the addition of GB could reduce the density, but decreased the flame retardancy of PP composites at the same time. To overcome this defect, ATH and mZrP with synergetic effect of flame retardancy were added into the composite. The dosage of each additive was optimized for achieving a balance of flame retardancy, good mechanical properties, and density. With 47 wt % ATH, 10 wt % GB, and 3 wt % mZrP, the peak heat release rate (pHRR) and total smoke production (TSP) of the composite **PP-4** were reduced by 91% and 78%, respectively. At the same time, increased impact strength was achieved compared with neat PP and the composite with ATH only. Maintaining the flame retardancy and mechanical properties, the density of composite **PP-4** (1.27 g·cm^−3^) is lower than that with ATH only (**PP-1**, 1.46 g·cm^−3^). Through this research, we hope to provide an efficient approach to designing flame retardant polypropylene (PP) composites with low density.

## 1. Introduction

In modern industry, such as architecture, electronic appliances, and vehicle manufacture, lightweight materials are highly demanded. Polypropylene (PP) is very promising in the areas mentioned above because of its low density (ca. 0.9 g·cm^−3^) [1,2]. However, the initial flammability of PP has severely limited its application in fields requiring high fire safety. Therefore, additive(s) which can simultaneously improve the flame retardancy and maintain the low density of the material show a high necessity. In recent years, the combination of metal hydroxides and phosphorus- and nitrogen-containing flame retardants has been considered as effective approaches to replace the traditional halogenated flame retardants which have bad reputations for environmental and health issues [3,4,5]. During the combustion, endothermic degradation and water releasing of metal hydroxides, such as aluminum trihydroxide (ATH), reduce the thermal release of the oxidizing reactions; at the same time, the thermal-stable ceramic-like protective layer formed prohibit the heat diffusion and isolate the burning material from the oxygen [6,7,8,9,10]. Most of time, the hydroxide additives increase the density of polymer composites, so searching for co-additives which can lower the density or even enhance the flame retardancy of the final composite is considered. In recent years, octacedylamine-modified α-zirconium phosphate (mZrP) has been reported as a synergetic flame-retardant additive of ATH with relatively low density (ca. 0.60–0.65 g·cm^−3^), which improves the flame retardant efficiency of composites by forming stronger intumescent char layers in the condensed phase [11,12,13,14,15]. On the other side, Glass Bubble (GB) is a cheap, easy-processing filler with even lower density (≤0.60 g·cm^−3^), which has been used in the production of low-density polyethylene foams [16,17,18]. To our knowledge, the influence of GB toward the flame retardancy and mechanical performances of PP has not been investigated yet. In our preliminary investigation, a certain range of ATH and GB dosages has been found with better flame retardancy and density decrease.

Herein, a series of PP composites have been prepared to investigate the differences in their fire-resistant performances and mechanical properties. The investigation has been developed by introducing mZrP into the PP/ATH/GB system. The dosage was optimized to get the formulation which endows the composite with balanced flame-retardant efficiency, good mechanical properties, and low material density. The material density, thermal stability, flame retardancy, and mechanical properties of the PP composites were characterized.

## 2. Materials and Methods 

PP, (REPSOL ISPLEN 070G2M) was supplied by Repsol YPF S.A. (Madrid, Spain) with density of 0.905 g·cm^−3^. Aluminum hydroxide, alumina martinal ol 104 leo was supplied by Quimidroga (Barcelona, Spain) with density 2.40 g·cm^−3^. Octacedylamine-modified α-zirconium phosphate (mZrP, 57% organic) with density 0.63 g·cm^−3^ was supplied by Prolabin & Tefarm (Perugia, Italy). Soda-lime borosilicate glass bubbles were supplied by 3M company (Maplewood, MN, USA) with density of 0.46–0.60 g·cm^−3^, and diameter of 15–65 μm.

All materials were dried at 80 °C for 4 h before processing to remove all the water. PP, ATH, GB, and mZrP were prepared using a twin-screw extruder (KETSE 20/40 EC, Brabender, Duisburg, Germany) at 200 °C at 60 rpm. The extruded strands were cut into pellets and then the PP composites were injected with an injection molding machine (Arburg 320 C, Awans, Belgium) for further tests. The details of the formulations for PP composites are listed in Table 1.

The limiting oxygen index (LOI) values were measured by an oxygen index meter (FTT, Derby, UK) with dimensions of 130 × 6.5 × 3.2 mm^3^ according to ASTM D2863-13 at room temperature. Cone calorimetry was carried out by a cone calorimeter (FTT, Derby, UK), following the procedures in ISO5660-1 at a heat flux of 50 kW·m^−2^. Specimens (100 × 100 × 4 mm^3^) were wrapped with aluminum foil. The testing of each sample was performed in duplicate. 

Thermogravimetric analysis (TGA) was measured in a TA-Q50 (TA instrument, New Castle, DE, USA) with a heating rate of 10 °C·min^−1^ from 25 °C to 800 °C in air.

The morphology of samples was observed by an EVO-MA15 (Zeiss, Oberkochen, Germany) scanning electronic microscopy (SEM). The surface of the samples was cover with a gold layer for conduction of the electrons.

The tensile strength and elongation at break were measured on an Instron Universal Tester machine (model 3384, Norwood, MA, USA) in accordance of ASTM D638-14 standard.

Charpy impact strength was measured on a universal experimental machine (ZORN, Stendal, Germany) in accordance with the procedures in ISO179-1 at room temperature. The size of specimens is 100 × 13 × 6.3 mm^3^. All the measurements were repeated ten times, from which the average value was taken, and the standard deviation of each test is reported.

DMA analysis was performed by a DMA Q800 Dynamic Mechanical Analyzer (TA Instruments, New Castle, DE, USA). PP samples with nominal dimensions of 17 × 6 × 3 mm^3^ were mechanically tested under two-point bending mode, with an amplitude range of 30 μm and frequency of 1 Hz. The samples were heated from −80 °C to 140 °C at a linear rate of 3 °C·min^−1^. 

## 3. Results and Discussion

### 3.1. Morphology Analysis

**Figure 1:**
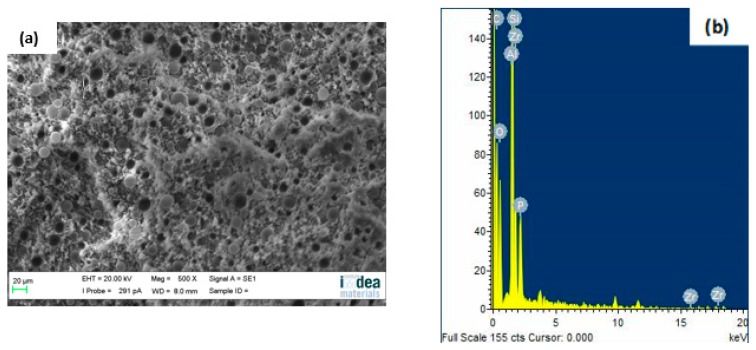
(**a**) Scanning electronic microscopy (SEM) image and (**b**) Energy dispersive X-ray spectroscopy (EDS) spectrum of **PP-4**.

**Figure 2:**
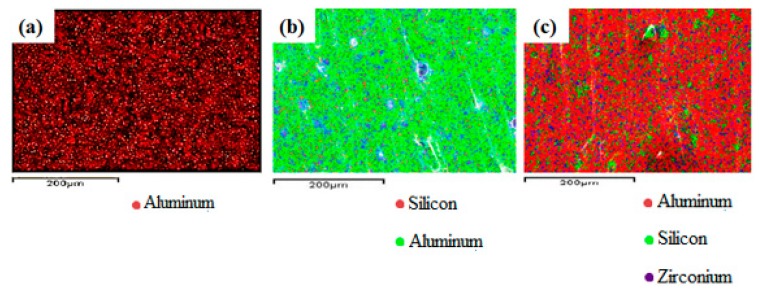
EDS results of (**a**) **PP-1**; (**b**) **PP-3**; (**c**) **PP-4**.

The dispersion of GB in the PP matrix was characterized by SEM. It can be seen from Figure 1a that spherical GBs are well dispersed in the polymer matrix. Figure 1b shows the energy dispersive X-ray spectroscopy (EDS) results of **PP-4**, the corresponding positions of the main peaks reflect the main composition of C, O, and Si in GBs, and other elements that correspond with ATH and mZrP, such as Al, Zr, and P, can also be observed. As Al, Si, and Zr are the representative elemental components in ATH, GB, and mZrP, respectively, the distribution of the three elements in PP composites can indicate the dispersion of the additives in the PP matrix. As shown in Figure 2, the dispersion of each element in **PP-1**, **PP-3**, and **PP-4** suggested the uniform distribution of ATH, GB, and mZrP in the PP matrix. 

### 3.2. Density of Composites

As shown in Table 1, the addition of ATH increases the density of the PP matrix from 0.89 g·cm^−3^ to 1.46 g·cm^−3^. With the addition of 10 wt % GB, the density of **PP-3** decreases by 21% compared with that of **PP-1** due to the low density of GBs (0.46–0.60 g·cm^−3^). The different densities of **PP-3** to **PP-6** indicated that apart from the GB, the ratio of ATH and mZrP is also a factor influencing the density of the composites. With the addition of 9 wt % mZrP, **PP-6** showed a lowest density (1.11 g·cm^−3^).

### 3.3. Thermal Stability of Fillers and Composites

**Figure 3:**
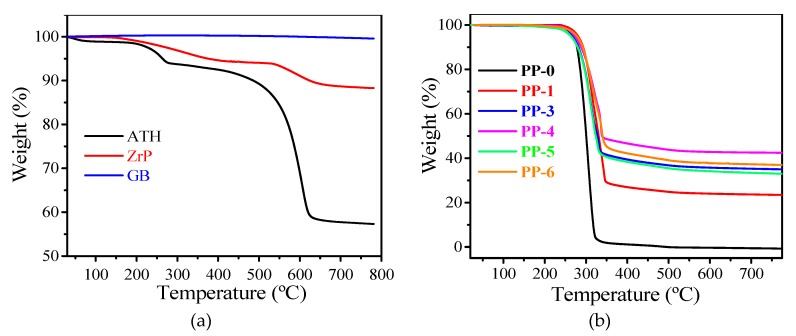
Thermogravimetric analysis (TGA) curves of (**a**) additives and (**b**) PP composites.

The thermal stability of neat PP (**PP-0**) and its composites (**PP-1** to **PP-6**) was studied by TGA. It can be seen in Figure 3b that the thermal degradation of all samples exhibits a one-step process which starts at ca. 230 °C. As expected, the thermal degradation curve of **PP-0** shows the biggest slope with the lowest temperatures of maximum rate of degradation (*T*_max_). The total degradation of **PP-0** can be found at ca. 340 °C. With the addition of ATH, the *T*_max_ of composites increased by more than 10 °C, which should ascribe to the high decomposition temperature of the dehydrated product of ATH (ca. 575 °C). Among samples with fillers, no big differences in the increment of *T*_max_ can be observed. However, the fillers in polymer composites improve the char residue significantly compared with that of **PP-0**. The char residue of **PP-1** is improved up to 23 wt %, which can be attributed to the degradation product of ATH, mainly Al_2_O_3_. Introduction of GB gives a further increase of the char residue amount [19] (35 wt % for **PP-3**). It can be observed in Figure 3a that GB has high thermal stability with a decomposition temperature higher than 800 °C, which implies that the thermal-stable GB could also be responsible for the increased char residue in the PP composites. The introduction of mZrP in to the PP matrix also increases the char residue (42 wt % for **PP-4**) by 95% and 45% compared with those of **PP-0** and **PP-1**. 

### 3.4. Fire-Resistant Behaviors

#### 3.4.1. Limiting Oxygen Index (LOI)

**Figure 4:**
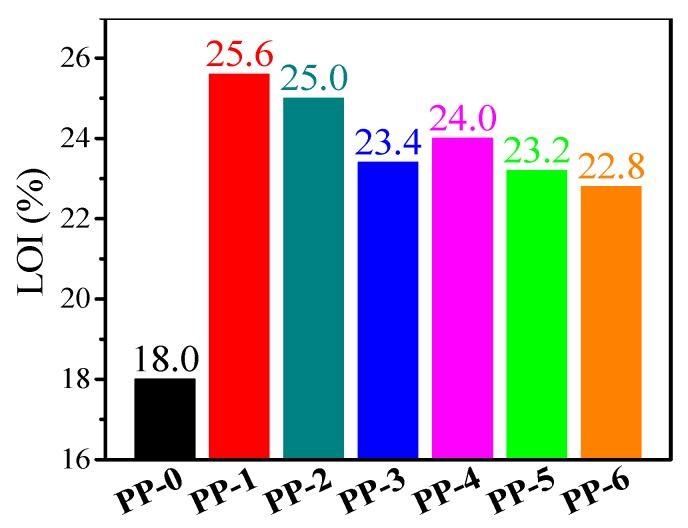
LOI results of PP composites.

Figure 3 exhibits the LOI results for neat PP and its composites. The LOI value of **PP-0** is 18.0%, which means that the neat PP easily catches fire in air. The LOI of **PP-1** increased up to 25.6% due to the flame-retardant effect of ATH. Without the participation of mZrP, the increasing dosage of GB decreased the LOI (23.4 for **PP-3** and 25.0 for **PP-2**). The addition of mZrP with 3 wt % (**PP-4**) increased the LOI from 23.4 (**PP-3**) to 24.0 due to the synergistic effect between ATH and mZrP. When the dosage of mZrP continuously increases (**PP-5** and **PP-6**), the LOI value decreases slightly but still stays higher than that of **PP-0** (Figure 4).

#### 3.4.2. Cone Calorimetry

For fire properties of polymers, the most effective laboratory evaluation is cone calorimetry, because the results can predict the combustion behavior of materials in real fire scenarios. The heat release rate (HRR) curves of neat PP and flame-retardant PP composites are shown in Figure 3. It can be seen that neat PP burns intensely, with a high peak of 1467 kW·m^−2^ in the HRR curve (pHRR). With 60 wt % ATH (**PP-1**), the pHRR is reduced to 280 kW·m^−2^ due to the endothermic decomposition of ATH, which releases water and produces an isolation barrier of aluminum oxide [20]:

2Al(OH)_3_ → Al_2_O_3_ + 3H_2_O (∆*H* = 1050 kJ·kg^−^^1^)

By maintaining the total additive content (60 wt %), 5 wt % GB has been introduced into the formulation (**PP-2**), pHRR value decreases to 212 kW·m^−2^, which can be attributed to the vitreous structure layer of GB formed on the surface of the polymer. This vitrified layer acts as a physical insulating layer between the gas phase and the condensed phase [21]. By further increasing GB to 10 wt %, pHRR of **PP-3** furtherly deceases to 190 kW·m^−2^. The suppression of heat release arises from ATH and GB is also confirmed by the significantly reduced maximum average rage of heat release (MARHE) values (Table 2). It has to be pointed out that a bimodality can be observed in the HRR curves of **PP-2** and **PP-3**, in which mZrP is absent. This phenomenon indicated the fragility of the char layer formed by the vitreous structure and oxide derived from ATH and GB.

On the other side, a non-linear change of the pHHR value can be found with addition of mZrP. For **PP-4** with 3 wt % mZrP, the pHHR turned out to be 136 kW·m^−2^, which is decreased by 28% and 91% compared with those of **PP-3** and **PP-0**, respectively. This performance can be attributed to synergetic effects between ATH and mZrP. During the combustion, mZrP can react with ATH to form a ceramic-like aluminophosphate compound, which may promote the formation of compact char layers efficiently, which hinders the diffusion of oxygen and flammable volatile products [22]. At the same time, during the decomposition of mZrP, phosphorus monoxide (PO·) species can quench active radicals in the gaseous phase [15]. However, higher pHRR values are found in samples with higher content of mZrP. In **PP-5** (mZrP 6 wt %) and **PP-6** (mZrP 9 wt %), pHRR values increase to 152 kW·m^−2^ and 189 kW·m^−2^, respectively, which implies that more than 3 wt % mZrP decreased the synergistic effect with ATH. This might be ascribed to the reduction of the main flame retardant (ATH). Except for neat PP, all the other samples (**PP-1** to **PP-6**) in cone calorimetry show very close effect heat of combustion (EHC), which means that no obvious variation of the gaseous phase mechanism can be found with the tuning of the composite formulation. The better flame retardancy presented by **PP-4** should be attributed to the improved condensed phase mechanism. The addition of GB shows a different influence toward the flame retardancy of the polymer composites in LOI test and the cone calorimetry. In cone calorimetry, **PP-2** and **PP-3** show better flame-retardant behaviors than **PP-1**, while in LOI the results show the opposite trend. The reason for this phenomenon should be assigned to different mechanisms of these characterizations. The shape of specimen in cone test is horizontal instead of vertical ones for LOI. Being placed in a vertical way, a “candlewick effect” might be promoted by GB so that specimens are flammable in LOI test [14], which can explain the decrease of LOI value of **PP-2** and **PP-3** compared with that of **PP-1**.

**Figure 5:**
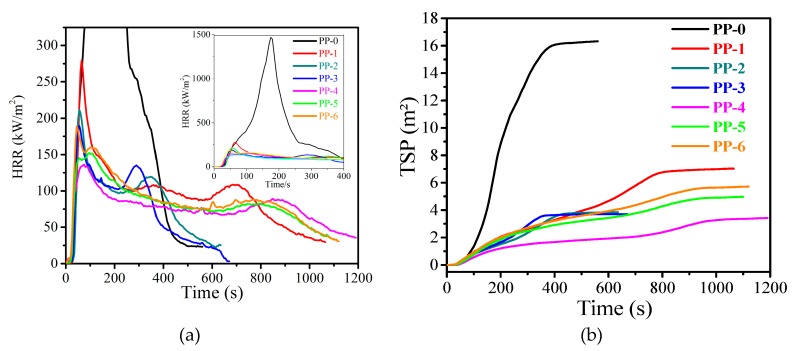
(**a**) Heat release rate (HRR) and (**b**) total smoke production (TSP) curves of PP composites

Smoke is one of the most important parameters in a real fire scenario. As shown in Figure 5b, the sample which has only ATH (**PP-1**) leads to an impressive 57% reduction in the total smoke release (TSR) due to the flame-retardant effect of ATH. With the addition of 5 wt % GB, **PP-2** shows a TSR of 433 m^2^·m^−2^. When increasing the amount of GB to 10 wt % (**PP-3**), the TSR goes down to 416 m^2^·m^−2^. Compared with **PP-0** and **PP-1**, the introduction of 10 wt % GB decreases TSR by 77% and 48%, respectively, which might be explained by a vitreous barrier effect of GB that blocks the distribution of soot and decreases the smoke produce [23]. The smoke produced is further reduced by mZrP introduced into the PP matrix. **PP-4** showed the lowest value of TSR (386 m^2^·m^−2^) with a reduction of 81% and 8%, respectively, to those of **PP-0** and **PP-3**. On the other hand, the TSR of **PP-5** and **PP-6** goes up to 561 m^2^·m^−2^ and 645 m^2^·m^−2^ with the increasing content of ATH. The changing trend is similar to the results of pHRR, meaning that a high content of mZrP has a negative effect on the flame-retardant system.

#### 3.4.3. Char Residue Analyses

**Figure 6:**
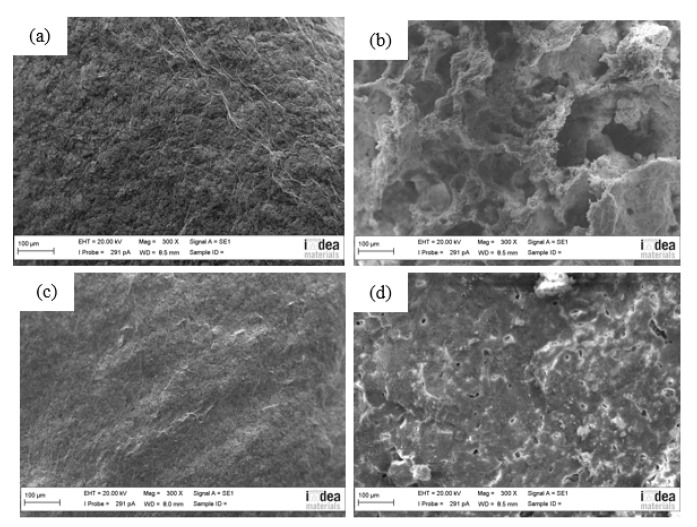
SEM images of charred residue of flame-retardant PP composites: (**a**) **PP-1**; (**b**) **PP-3**; (**c**) **PP-4**; (**d**) **PP-6**.

Figure 6 shows SEM images of char residue of PP composites after cone calorimetry. It can be seen from Figure 6a that a compact and homogenous char residue has been formed on the surface of **PP-1** after combustion, which might be caused by the decomposition of ATH at high temperature, which forms aluminum oxide as a tight layer on the surface of matrix [24]. The layer acts as a good thermal insulation layer between the gas phase and condensed phase, protecting the matrix from further combustion. However, the incorporation of GB (**PP-3**) significantly decreases the integrity of the protect layer (Figure 6b). The layer on the surface of burnt **PP-3** is loose, together with a great amount of big holes inside, indicating the negative influence of GB toward the char morphology of the composite. The morphology of char residue of PP-3 provides a more liable explanation of the bimodality HRR curves. Figure 6c corresponding to **PP-4** shows a compact and homogenous char layer which appears even more condensed than that of **PP-1**, suggesting that the synergetic working of mZrP with ATH can greatly improve the quality of the char layer under the proper dosage ratio. In Figure 6d, it can be seen that more small pores are formed on the surface of the char layer of **PP-6** compared with that of **PP-4**. The morphology of the char residue also provides a side-proof for the different flame retardancy of PP composites with different mZrP ratios.

### 3.5. Dynamic Analysis of Mechanics

DMA was used to evaluate the effects of all the additives toward the thermomechanical properties of PP matrix because of its sensitivity to the relaxation behavior of materials and the behaviors of materials against a sinusoidal stress. The mechanical relaxation data of the composites are depicted in the form of storage modulus (*E*′) and loss factor (tan*δ*), respectively.

**Figure 7:**
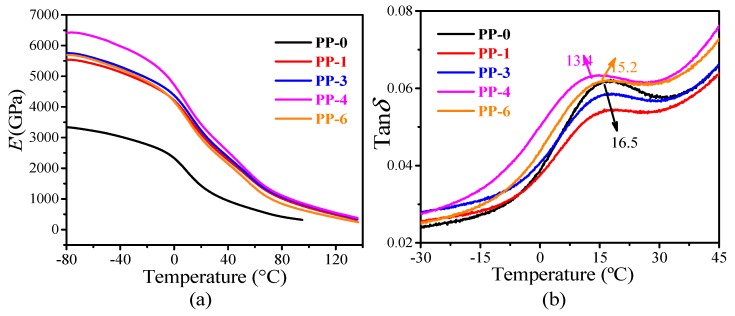
(**a**) Storage modulus and (**b**) glass transition temperature of PP composites

Figure 7a shows the variations of *E*′ of neat PP and those composites as a function of temperature. Firstly, it can be seen that all PP composites had higher *E*′ values than neat PP due to the reinforcement effect of inorganic fillers. Moreover, among the PP composites with the same filler content, **PP-4** displayed the maximum value of *E*′. It should be pointed out that both **PP-4** and **PP-6** contained three different fillers, so the better performance of **PP-4** might be attributed to the synergistic enhancement effect of the fillers with proper addition amount. The main storage modulus drop is observed at ca. 10 °C due to *β* relaxation as it is described in tan*δ*. This relaxation is associated with the glass transition of the amorphous region of PP, known as the major transition in the composites [25]. Under glass transition temperature, the value of *E*′ decreases as the temperature increases, indicating the glass/rubber transition behavior of the composites [26].

Figure 7b shows the variation of tan*δ* along with the temperature increase. For **PP-0**, **PP-1**, and **PP-3**, the maximum value of tanδ presented at approximately 16.5 °C, which indicates that the incorporation of ATH and GBs has insignificant impact to the *T*_g_ of PP matrix. In **PP-4**, with further addition of mZrP into the composite, *T*_g_ decreased to 13.4 °C. A similar phenomenon has been found in epoxy resin/SiO_2_ system, in which a decrease of *T*_g_ was detected after the addition of mZrP [27]. In **PP-6**, a slightly increased *T*_g_ was found (15.2 °C), which is still lower than that of **PP-0**. This result suggested that the content of mZrP is an important factor influencing the polymer–filler interaction, which might significantly influence the mobility of the polymer chains.

### 3.6. Mechanical Properties

Tensile strength, Young’s modulus and impact strength were adopted to characterize the influence of fillers toward the mechanical properties of PP polymer composites. Table 3 shows that **PP-0** has the highest tensile strength (34.7 MPa). With the addition of ATH, the tensile strength of **PP-1** remarkably decreases by 41% compared with that of **PP-0**. The introduction of GB further decreases the tensile strength down to 18.3 MPa. It is widely reported that rigid fillers will increase the rigidity of polymer matrices. As displayed in this work, the composite with a proper amount of ATH and mZrP can achieve a similar tensile strength (20.2 MPa for **PP-4**) to that of **PP-1**. When the mZrP amount is more than 3 wt %, the tensile strength decreases.

It can be observed from Table 3 that the addition of ATH exhibits negligible influence on the impact strength of the composite, just as shown in the impact strength of **PP-1**. Nevertheless, considering the impact strength of **PP-3** which simply contains ATH and GB, we can see that with the participation of GB, the composite becomes more brittle by decreasing the impact strength by ca. 25% compared with that of **PP-0** and **PP-1**, which could be possibly explained with the PP chains stiffened by the glassy structure of GB so that the impact energy cannot be dissipated with any movement. Nevertheless, a certain dosage of mZrP (ca. 3 wt %) overcomes the drawbacks of GB so that the higher impact strength value can be achieved in **PP-4**, probably because the mZrP, as a rigid filler decorated with the polymer affinitive alkyl subgroups, plays a reinforcing role in the composite.

## 4. Conclusions

In this work, flame-retardant polypropylene (PP) composites with relatively low density were prepared with ATH, GB, and mZrP as additives. The density, morphology, flame retardancy, thermal stability, and mechanical properties were characterized in the composites. The results show that the fillers dispersed homogenously in the PP matrix and the addition of GB decreased the density of PP composites effectively. However, the flame retardancy was also deteriorated by the addition of GB. To solve this problem, mZrP was introduced into the composites to improve flame retardancy, and the corresponding ratio of each additive was optimized. In order to achieve the balance of flame retardancy, mechanical properties, and density, an appropriate ratio of ATH, GB, and mZrP was investigated. With the addition of 47 wt % ATH, 10 wt % GB, and 3 wt % mZrP, the pHRR and TSP of the composite (PP-4) were reduced by 91% and 81% compared with neat PP, respectively. The morphology study of the char residue also provided evidence for the improvement of the flame retardancy of PP composites. Furthermore, the mechanical properties of **PP-4** were comparable with that of flame-retardant PP composites with only ATH. Through this research, an efficient approach for the design of flame-retardant polypropylene (PP) composites with low density was provided.

## Figures and Tables

**Table 1:** Formulation and density of polypropylene (PP) composites.

Sample	PP	ATH	GB	mZrP	Density (g·cm^−3^)
**PP-0**	100	0	0	0	0.89
**PP-1**	40	60	0	0	1.46
**PP-2**	40	55	5	0	1.19
**PP-3**	40	50	10	0	1.16
**PP-4**	40	47	10	3	1.27
**PP-5**	40	44	10	6	1.14
**PP-6**	40	41	10	9	1.11

**Table 2:** Cone calorimetry of PP composites.

Sample	TTI(s)	pHRR (kW·m^−2^)	THR (MJ·m^−2^)	TSP (m^2^)	TSR (m^2^·m^−^^2^)	MARHE (kW/m^2^)	EHC (MJ/kg)
PP-0	32	1470	175	16.3	1845	573	46
PP-1	34	280	98	7.0	795	139	30
PP-2	31	212	53	3.9	433	107	28
PP-3	36	190	49	3.7	416	112	27
PP-4	24	136	90	3.4	386	98	29
PP-5	24	152	91	4.9	561	114	30
PP-6	21	189	98	5.7	645	131	32

**Table 3:** Mechanical properties of the PP composites.

Sample	Tensile Strength (MPa)	Young’s Modulus (MPa)	Impact Strength (kJ·m^−2^)
PP-0	34.7 ± 1.1	1312 ± 61	3.9 ± 0.1
PP-1	20.6 ± 0.6	1982 ± 83	4.0 ± 0.5
PP-3	18.3 ± 1.0	2552 ± 69	3.1 ± 0.4
PP-4	20.2 ± 3.8	2190 ± 198	4.3 ± 0.9
PP-5	16.9 ± 0.8	2074 ± 119	3.3 ± 0.2
PP-6	15.1 ± 0.5	2308 ± 13	3.3 ± 0.6
